# Brain region-specific lesions in the early AIDS monkey model

**DOI:** 10.1093/lifemedi/lnag033

**Published:** 2026-09-16

**Authors:** Tian-Zhang Song, Yan Hu, Shi-Zhe Wang, Huan-Zhi Chen, Bei-Bei Tang, Jin-Nan Li, Qing Li, Rong-Yao Huang, Li-Rong Zhang, Lin Xu, Yong-Tang Zheng

**Affiliations:** State Key Laboratory of Genetic Evolution & Animal Models, Kunming Institute of Zoology, Chinese Academy of Sciences, Kunming 650201, China; Key Laboratory of Bioactive Peptides of Yunnan Province, KIZ-CUHK Joint Laboratory of Bioresources and Molecular Research in Common Diseases, Kunming Institute of Zoology, Chinese Academy of Sciences, Kunming 650223, China; State Key Laboratory of Genetic Evolution & Animal Models, Kunming Institute of Zoology, Chinese Academy of Sciences, Kunming 650201, China; Key Laboratory of Bioactive Peptides of Yunnan Province, KIZ-CUHK Joint Laboratory of Bioresources and Molecular Research in Common Diseases, Kunming Institute of Zoology, Chinese Academy of Sciences, Kunming 650223, China; Medical School, University of Chinese Academy of Sciences, Beijing 101408, China; State Key Laboratory of Genetic Evolution & Animal Models, Kunming Institute of Zoology, Chinese Academy of Sciences, Kunming 650201, China; Medical School, University of Chinese Academy of Sciences, Beijing 101408, China; Key Laboratory of Animal Models and Human Disease Mechanisms of Yunnan Province, Laboratory of Learning and Memory, Kunming Institute of Zoology, Chinese Academy of Sciences, Kunming 650223, China; Medical School, University of Chinese Academy of Sciences, Beijing 101408, China; State Key Laboratory of Genetic Evolution & Animal Models, Kunming Institute of Zoology, Chinese Academy of Sciences, Kunming 650201, China; Key Laboratory of Bioactive Peptides of Yunnan Province, KIZ-CUHK Joint Laboratory of Bioresources and Molecular Research in Common Diseases, Kunming Institute of Zoology, Chinese Academy of Sciences, Kunming 650223, China; KIZ-SU Joint Laboratory of Animal Model and Drug Development, College of Pharmaceutical Sciences, Soochow University, Suzhou 215021, China; State Key Laboratory of Genetic Evolution & Animal Models, Kunming Institute of Zoology, Chinese Academy of Sciences, Kunming 650201, China; Key Laboratory of Animal Models and Human Disease Mechanisms of Yunnan Province, Laboratory of Learning and Memory, Kunming Institute of Zoology, Chinese Academy of Sciences, Kunming 650223, China; State Key Laboratory of Genetic Evolution & Animal Models, Kunming Institute of Zoology, Chinese Academy of Sciences, Kunming 650201, China; Key Laboratory of Bioactive Peptides of Yunnan Province, KIZ-CUHK Joint Laboratory of Bioresources and Molecular Research in Common Diseases, Kunming Institute of Zoology, Chinese Academy of Sciences, Kunming 650223, China; State Key Laboratory of Genetic Evolution & Animal Models, Kunming Institute of Zoology, Chinese Academy of Sciences, Kunming 650201, China; National Research Facility for Phenotypic and Genetic Analysis of Model Animals (Primate Facility), National Resource Center for Non-Human Primates, Kunming Institute of Zoology, Chinese Academy of Sciences, Kunming 650107, China; State Key Laboratory of Genetic Evolution & Animal Models, Kunming Institute of Zoology, Chinese Academy of Sciences, Kunming 650201, China; Key Laboratory of Bioactive Peptides of Yunnan Province, KIZ-CUHK Joint Laboratory of Bioresources and Molecular Research in Common Diseases, Kunming Institute of Zoology, Chinese Academy of Sciences, Kunming 650223, China; KIZ-SU Joint Laboratory of Animal Model and Drug Development, College of Pharmaceutical Sciences, Soochow University, Suzhou 215021, China; State Key Laboratory of Genetic Evolution & Animal Models, Kunming Institute of Zoology, Chinese Academy of Sciences, Kunming 650201, China; Key Laboratory of Animal Models and Human Disease Mechanisms of Yunnan Province, Laboratory of Learning and Memory, Kunming Institute of Zoology, Chinese Academy of Sciences, Kunming 650223, China; State Key Laboratory of Genetic Evolution & Animal Models, Kunming Institute of Zoology, Chinese Academy of Sciences, Kunming 650201, China; Key Laboratory of Bioactive Peptides of Yunnan Province, KIZ-CUHK Joint Laboratory of Bioresources and Molecular Research in Common Diseases, Kunming Institute of Zoology, Chinese Academy of Sciences, Kunming 650223, China

**Keywords:** HIV-associated neurocognitive disorder, nonhuman primate model, subcortical nuclei, microglia, Tat protein

## Abstract

Brain lesions show pronounced regional heterogeneity during chronic or advanced human immunodeficiency virus (HIV) infection; however, the regional neuropathogenesis during the acute phase remains unclear. By establishing an acute simian immunodeficiency virus (SIV)-infection model in rhesus macaques, we found acute infection-induced robust neuroinflammation and neuronal damage. Compared with the cerebral cortex, subcortical nuclei displayed earlier and more severe neuronal degeneration and loss of Nissl bodies, accompanied by greater upregulation of immune-related genes. Although SIV RNA loads in cerebrospinal fluid (CSF) and plasma were strongly correlated and peaked during acute infection, brain tissues showed no evidence of early viral reservoir establishment. The viral transactivator Tat persisted in CSF and was broadly distributed throughout the brain via passive diffusion along perivascular spaces. Similar transcriptional and inflammatory profiles were observed between macaques with acute infection and mice receiving intracisternal Tat infusions identified Tat as a principal mediator of early SIV-induced neurotoxicity. Microglia in a primed immune-vigilant state exhibited considerable enrichment in subcortical nuclei, driving exaggerated responses to Tat exposure and resulting in region-specific neuropathological outcomes. Collectively, these findings delineate the spatiotemporal landscape of acute HIV/SIV-induced brain injury and establish viral Tat as a mechanistic driver of acute neuropathogenesis.

## Introduction

Human immunodeficiency virus (HIV)-associated neurocognitive disorder (HAND) affects 30%–50% of people living with HIV (PLWH), ranging from asymptomatic neurocognitive impairment and mild neurocognitive disorder to HIV-associated dementia [1, 2]. Although combination antiretroviral therapy (cART) has substantially reduced the incidence of severe dementia, the overall HAND prevalence remains largely unchanged due to the continued predominance of mild and asymptomatic cases [3].

In chronic or advanced HIV infection, brain lesions show pronounced regional heterogeneity: subcortical structures (e.g., basal ganglia, hippocampus) harbor more viral RNA, intact proviruses, and viral proteins than cortical regions [4–6]. Clinical imaging and histopathological data consistently show that subcortical nuclei undergo more severe and extensive damage than the cerebral cortex, characterized by region-specific blood–brain barrier (BBB) disruption, metabolic dysregulation, and structural degeneration [7–9]. Importantly, based on colocalization-likelihood estimation, Israel et al. [10] demonstrated that cortical atrophy correlated with HIV infection itself, whereas subcortical atrophy represented an independent risk factor for HAND.

Acute HIV infection also causes rapid functional and structural alterations in PLWH. Viral RNA appears in cerebrospinal fluid (CSF) as early as 8 days postinfection, and neuronal injury and immune activation (elevated neopterin and CXCL10) emerge within months after transmission [11]. Within the initial 12 weeks, over half of patients exhibit neurological symptoms (e.g., cognitive decline, motor impairment, peripheral neuropathy), and neuroimaging reveals cortical thinning and third ventricle expansion in acutely infected individuals [12, 13]. Notably, longitudinal analyses indicate that in long-term cART-treated individuals, brain lesions occur predominantly during the early untreated phase, with only minimal progression over subsequent years [14, 15]. However, due to the challenges of precisely defining the timing of transmission and acquiring cerebral samples during early infection, the regional dynamics and mechanisms of cerebral involvement in acute infection remain unclear.

Simian immunodeficiency virus (SIV)-infected nonhuman primates (NHPs) are the most relevant models for HIV neuropathogenesis. Highly passaged or genetically modified SIV strains, or *in vivo* depletion of CD4^+^ and CD8^+^ T lymphocytes, can rapidly induce neurological manifestations in NHPs. However, such manipulations typically accelerate progression to acquired immunodeficiency syndrome (AIDS), failing to accurately mirror the early neuropathological events of PLWH [16]. In contrast, SIVmac239 infection establishes a long-term progressive disease course, with approximately 25% of animals developing encephalitis within 1–3 years, making it a suitable model for studying early neuropathology [17, 18]. We previously established stable SIVmac239 infection in Chinese rhesus macaques (RMs, *Macaca mulatta*) and northern pig-tailed macaques (*Macaca leonina*) [19]. In the present study, pathological, virological, neuroimaging, and transcriptomic profiles were systematically characterized across distinct brain regions of RMs during acute SIVmac239 infection, focusing on comparative analyses between cortical areas (e.g., frontal, parietal, occipital, temporal, and insular cortices) and subcortical nuclei (e.g., caudate nucleus, putamen, globus pallidus, hippocampus, and thalamus).

## Results

### Rapid neuroinflammation and neural impairment during acute SIV infection

RMs were intravenously inoculated with 4000 median tissue culture infectious doses (TCID_50_) of SIVmac239. Viral dynamics were assessed by quantifying plasma viral loads (pVLs) and CSF viral loads (cVLs) in three infected RMs (Fig. 1A). Both pVLs and cVLs exhibited synchronous temporal profiles, with peak levels consistently detected at 2 weeks postinfection (wpi). Linear regression analysis demonstrated a strong positive correlation between cVLs and pVLs, suggesting considerable homology between pVLs and cVLs during acute SIV infection. Biomarkers in CSF were further analyzed. Concentrations of neopterin, neurofilament light chain (NF-L), chitinase-3-like protein 1 (YKL-40), and albumin remained unchanged between 0 and 2 wpi (Fig. 1B), whereas cytokine levels showed a significant increase at 2 wpi (Fig. 1C). These findings suggest that inflammation, rather than neurodestruction, dominates the central nervous system (CNS) response during the early acute stage.

**Figure 1.**
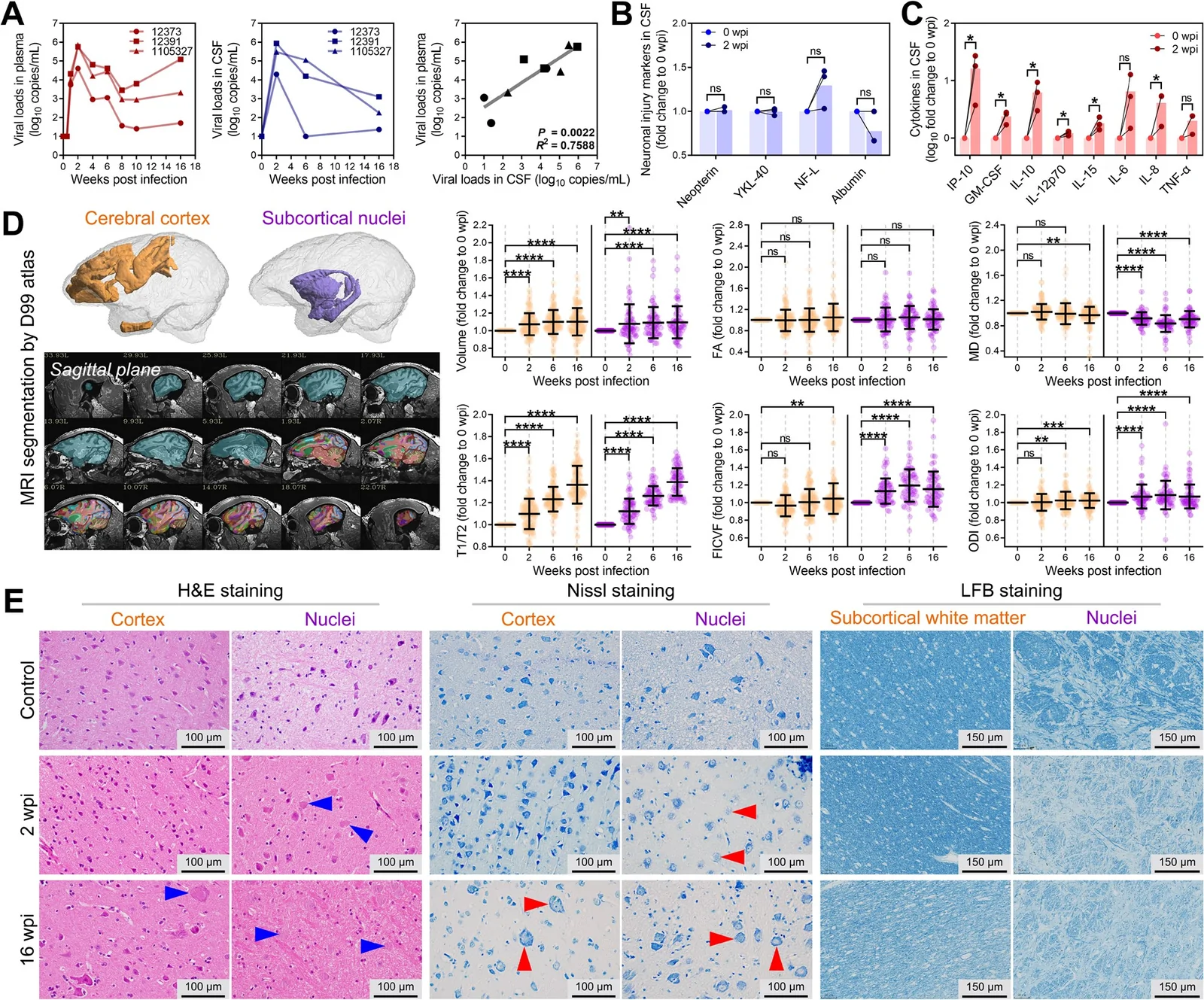
Viral dynamics, neuroinflammation, and neural impairment in rhesus macaques (RMs) with acute SIVmac239 infection. (A) SIVmac239 RNA loads in plasma (red) and cerebrospinal fluid (CSF, blue), and their linear correlation (black). (B) Fold changes of neurodestructive biomarkers, including neopterin, chitinase-3-like protein 1 (YKL-40), neurofilament light chain (NF-L), and albumin, in CSF at 2 weeks postinfection (wpi) relative to baseline (0 wpi). (*n* = 3 per time point; mean with individual values; paired *t*-tests). (C) Fold changes of cytokines (IP-10, GM-CSF, IL-10, IL-12p70, IL-15, IL-6, IL-8, TNF-α) in CSF at 2 wpi relative to 0 wpi. (*n* = 3 per time point; mean with individual values; paired *t*-tests). (D) Fold changes of structural MRI indices (volume and T1/T2 ratio) and neurite orientation dispersion and density imaging (NODDI)-derived metrics, including fractional anisotropy (FA), mean diffusivity (MD), intracellular volume fraction (FICVF), and orientation dispersion index (ODI), in 46 cortical and 23 subcortical regions (based on the D99 atlas, left panel) at 2, 6, and 16 wpi relative to 0 wpi. Each data point represents an individual brain region (orange: cortical regions; purple: subcortical regions) from three RMs per time point. (*n* = 138 and 69 for cortical and subcortical regions, respectively; mean ± SD; two-way ANOVA). (E) Representative histopathological staining, including hematoxylin and eosin (H&E), Nissl, and Luxol fast blue (LFB), in the cerebral cortex and the subcortical nuclei. Blue arrow: impaired nerve cells; red arrow: abnormal Nissl bodies. Scale bars: 100 μm (for H&E staining and Nissl staining) and 150 μm (for Luxol fast blue staining). (ns, not significant; ^*^P < 0.05, ^**^P < 0.01, ^***^P < 0.001, ^****^P < 0.0001).

To evaluate longitudinal cerebral alterations during acute SIV infection, magnetic resonance imaging (MRI) was performed at 0, 2, 6, and 16 wpi. Quantitative measurements of structural MRI indices and metrics derived from neurite orientation dispersion and density imaging (NODDI) were extracted from 46 cortical and 23 subcortical regions using the D99 macaque brain atlas (Fig. 1D). Significant increases in both volume and T1/T2 ratio were observed in cortical and subcortical regions at 2, 6, and 16 wpi compared with preinfection baselines, indicating rapid structural and microenvironmental changes during the acute stage. NODDI-derived metrics further demonstrated widespread microstructural damage (Fig. 1D). Specifically, the cerebral cortex showed significant alterations by 16 wpi, whereas the subcortical nuclei exhibited such changes as early as 2 wpi, characterized by unchanged fractional anisotropy (FA), reduced mean diffusivity (MD), elevated intracellular volume fraction (FICVF), and increased orientation dispersion index (ODI). These metric shifts are consistent with patterns of neuroinflammation/edema (unchanged FA and reduced MD), neuroinflammation (elevated FICVF), and axonal disorganization (elevated ODI), collectively indicating that microstructural neural impairment initiates in the subcortical nuclei during the early acute phase of SIV infection [20, 21].

Histological staining was employed to assess neuronal and myelin pathology during acute SIV infection. Both hematoxylin and eosin (H&E) and Nissl staining revealed no diffuse neuronal injury across brain regions during acute infection; however, localized neuronal degeneration, nuclear dissolution, and reduction or absence of Nissl bodies were sporadically detected in the cerebral cortex at 16 wpi and in the subcortical nuclei at 2 wpi and 16 wpi (Fig. 1E). Subsequently, Luxol Fast Blue (LFB) staining demonstrated significant demyelination in both subcortical white matter and nuclei at 16 wpi, with subcortical nuclei exhibiting preferential myelin loss at 2 wpi (Fig. 1E). Collectively, these data demonstrate that acute SIV infection induces rapid brain injury, with subcortical nuclei exhibiting earlier and more severe structural and histological alterations compared to the cerebral cortex.

### More severe immune- and neural-associated transcriptomic changes in the subcortical nuclei during acute SIV infection

Bulk RNA sequencing (RNA-seq) was performed on the ­cerebral cortex and subcortical nuclei collected from healthy and SIV-infected RMs (Table S1). Gene Ontology (GO) biological process and Kyoto Encyclopedia of Genes and Genomes (KEGG) enrichment analysis of differentially expressed genes (DEGs) revealed robust enrichment of immune-, defense- and infection-related terms/pathways in the cerebral cortex at 2 wpi compared to control (Fig. 2A). Comparison of 16 wpi vs. 2 wpi showed that several immune- and infection-related KEGG pathways remained enriched, indicating further immune modulation during the later acute phase (Fig. 2A). In the subcortical nuclei, similar trends of immune-, defense-, and infection-related terms/pathways enrichment were observed at 2 wpi relative to control (Fig. 2B). Notably, at 16 wpi, DEGs in the subcortical nuclei exhibited pronounced enrichment in pathways related to neurodegeneration and synaptic dysfunction, including amyotrophic lateral sclerosis, Huntington’s disease, and neuroactive ligand–receptor interaction (Fig. 2B).

**Figure 2.**
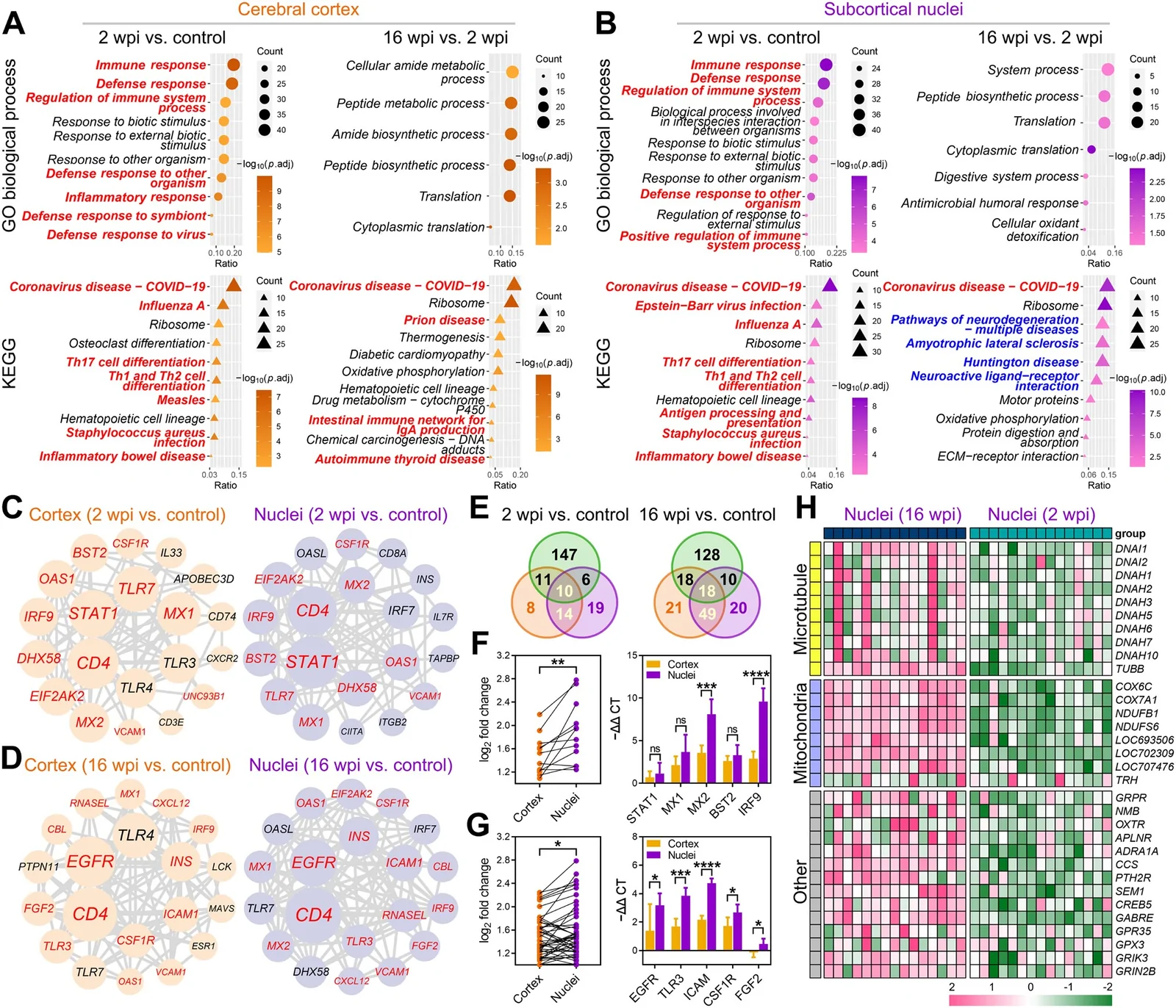
Transcriptomic profiling in the cerebral cortex and subcortical nuclei during acute SIVmac239 infection. (A, B) Top 10 enriched Gene Ontology Biological Process (GO biological process) terms (upper panel) and top 10 enriched Kyoto Encyclopedia of Genes and Genomes (KEGG) pathways (lower panel) among differentially expressed genes (DEGs) in the cerebral cortex (A) and subcortical nuclei (B) for two comparisons: 2 wpi vs. control, and 16 wpi vs. 2 wpi. Red indicates immune-, defense-, or infection-related terms/pathways; blue indicates nerve-related terms/pathways. (C, D) Protein–protein interaction (PPI) networks of the top 20 upregulated immune-related DEGs (ranked by degree) for two comparisons: 2 wpi vs. control (C), and 16 wpi vs. control (D). Red: co-upregulated in both cerebral cortex and subcortical nuclei; black: upregulated in only one of the two regions. (E) Venn diagrams showing overlaps among three sets of immune-related DEGs: upregulated in cerebral cortex during SIV infection (orange), upregulated in subcortical nuclei during SIV infection (purple), and differentially expressed between subcortical nuclei and cerebral cortex in healthy controls (green). (F, G) Co-upregulated immune-related gene analysis between cerebral cortex and subcortical nuclei for two comparisons: 2 wpi vs. control (F), and 16 wpi vs. control (G). Left panel shows fold change of shared upregulated—immune-related DEGs, excluding region-differential genes identified in healthy controls (*n* = 12 and 47 shared DEGs for F and G, respectively; individual values; paired *t*-tests). Right panel shows qPCR validation of hub gene expression levels (*n* = 5 per group; mean ± SD; unpaired *t*-tests). (H) Heatmap of DEGs associated with nerve-related biological processes in subcortical nuclei (16 wpi vs. 2 wpi). (ns, not significant; ^*^P < 0.05, ^**^P < 0.01, ^***^P < 0.001, ^****^P < 0.0001).

To further analyze transcriptomic differences between the cerebral cortex and subcortical nuclei, we first focused on immune-related DEGs, the majority of which were upregulated during SIV infection. Using protein–protein interaction (PPI) networks of the top 20 upregulated immune-related DEGs, we found that the cerebral cortex and subcortical nuclei shared similar hub genes at both time points compared to control. At 2 wpi, common hub genes included *STAT1*, *CD4*, *MX1*, *DHX58*, and *BST2* (Fig. 2C); at 16 wpi, shared hub genes included *EGFR*, *CD4*, *CSF1R*, *INS*, and *ICAM1* (Fig. 2D). Subsequently, using Venn diagrams, we identified co-upregulated immune-related DEGs between the cerebral cortex and subcortical nuclei. At 2 wpi vs. control, 14 genes were co-upregulated; at 16 wpi vs. control, 49 genes were co-upregulated, after excluding genes that showed baseline differences between the two regions in healthy controls (Fig. 2E). Notably, these co-upregulated DEGs showed greater fold changes in subcortical nuclei than in the cerebral cortex at both time points. Specifically, *MX2* and *IRF9* were more highly expressed in subcortical nuclei at 2 wpi (Fig. 2F), whereas *EGFR*, *TLR3*, *ICAM1*, *CSF1R*, and *FGF2* showed higher expression at 16 wpi (Fig. 2G), indicating stronger antiviral immune responses in subcortical nuclei during early acute infection. Heatmaps of DEGs associated with nerve-related biological processes in subcortical nuclei (16 wpi vs. 2 wpi), showed that many of these genes were associated with microtubule organization (e.g., *DNAI1*, *DNAH1*, and *TUBB*) and mitochondrial function (e.g., *COX6C* and *NDUFS6*), suggesting either ongoing pathology or compensatory axonal remodeling (Fig. 2H). Together, these transcriptomic analyses reveal that the subcortical nuclei not only mount a stronger immune response but also show signs of neuronal injury and axonal damage, underscoring their heightened vulnerability during acute SIV infection.

### Viral proteins rather than viral particles penetrate cerebral tissues during acute SIV infection

To assess viral dissemination within these compartments, viral RNA, DNA, and proteins were quantified in cerebral and peripheral tissues using qPCR and Western blotting. Neither viral RNA nor DNA was detected in the cerebral cortex or subcortical nuclei at 2 wpi and 16 wpi, in contrast to robust signals in peripheral tissues such as the spleen, colon, and liver (Fig. 3A and 3B). Consistently, the Gag precursor protein p55, essential for virion assembly, was undetectable in cerebral regions at either time point (Fig. 3A and 3B).

**Figure 3.**
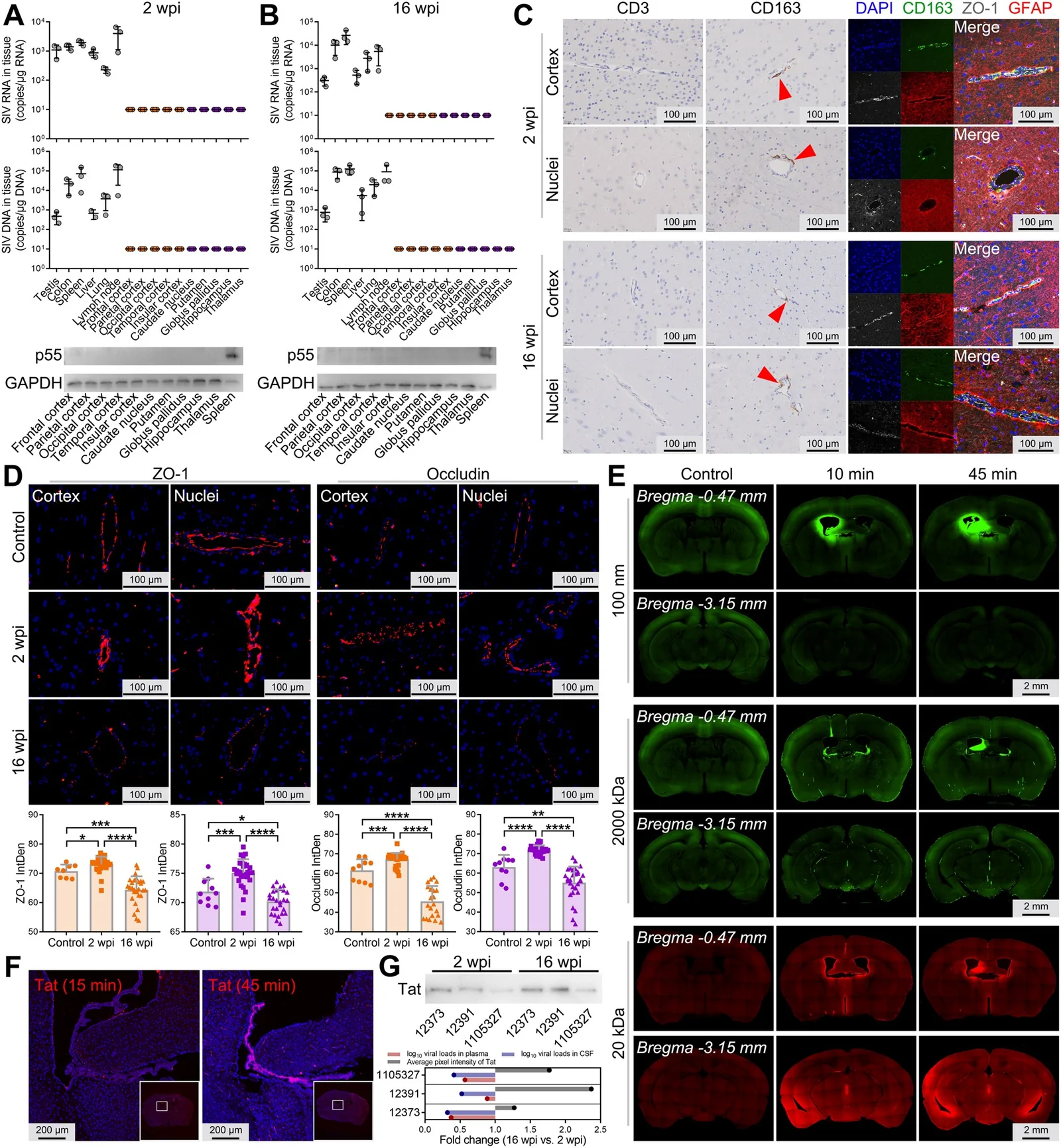
Viral entry and infection in cerebral tissues during acute SIV infection. (A, B) Quantification of SIVmac239 RNA, DNA, and p55 protein in peripheral tissues and cerebral tissues at 2 wpi (A) and 16 wpi (B). (C) Representative immunohistochemical staining of cerebral tissues. Left: conventional staining for CD3 (lymphocytes) and CD163 (macrophages). Right: multiplex staining for CD163, ZO-1, and GFAP. Red arrow: perivascular macrophages. (D) Representative immunofluorescence images (upper panel) and quantitative analysis of fluorescence intensity (lower panel) for tight junction proteins ZO-1 and occludin in the cerebral cortex (orange) and subcortical nuclei (purple) of RMs. (*n* values for the four panels [Control/2 wpi/16 wpi] are 8/30/29, 10/29/27, 10/30/21, and 10/30/30, respectively; mean ± SD; unpaired *t*-tests). (E) Fluorochrome infusion into the right lateral ventricle of mice. Shown are 100 nm FluoSpheres, 2000 kDa FITC-dextran, and 20 kDa TRITC-dextran. The two control groups in Fig. 3E (100 nm and 2000 kDa) are representative images from the same set of images. (F) Immunofluorescence staining of HIV-1 Tat in mouse brain tissues at 15 and 45 min following intracerebroventricular Tat infusion. (G) Quantification of SIV Tat protein in CSF. Upper panel: Western blot analysis of SIV Tat protein in CSF of SIV-infected RMs at 2 wpi and 16 wpi. Lower panel: fold changes in plasma and CSF viral loads (log_10_) and average pixel intensity of Tat at 16 wpi relative to 2 wpi. (^*^P < 0.05, ^**^P < 0.01, ^***^P < 0.001, ^****^P < 0.0001).

To investigate potential viral entry routes, cerebral sections were examined for peripheral immune cell infiltration. Immunohistochemistry targeting CD3 and CD163 revealed an absence of lymphocyte migration into the parenchyma, and macrophage infiltration remained restricted to the perivascular spaces (PVS) delineated by astrocytic endfeet (Fig. 3C). These findings argue against significant parenchymal viral entry via infected immune cells (Trojan horse mechanism) during the acute phase, as HIV-1 infection cannot be effectively established in astrocytes via cell-free or cell-to-cell routes [22]. Further analysis of BBB integrity showed that expression of the tight junction proteins ZO-1 and occludin was elevated at 2 wpi but declined by 16 wpi (Fig. 3D), suggesting that the BBB is not overtly ­disrupted during the early acute stage.

To resolve the apparent discrepancy between high cVLs, absent brain parenchymal infection, and robust STAT1-driven antiviral responses in cerebral tissues, a tracer-based simulation was conducted in mice via intracerebroventricular infusion (Fig. 3E). Fluorescent polystyrene microspheres (100 nm), approximating the size of intact HIV/SIV virions, failed to ­traverse the PVS or contralateral ventricle, confirming limited particle mobility in CSF. In contrast, smaller molecular weight dextrans (20 kDa and 2000 kDa) exhibited differential diffusion, with only the low-molecular-weight tracer penetrating brain parenchyma. These data imply that soluble viral proteins below 20 kDa can diffuse from the CSF into brain tissue through PVS pathways. Tat is regarded as a major neurodestructive ­factor in HAND [23]. Given its low molecular weight (14 kDa–16 kDa), HIV-1 Tat was evaluated for diffusion potential. Intracerebroventricular infusion of recombinant Tat confirmed its delivery to cerebral parenchyma and PVS (Fig. 3F), and Western blotting of CSF samples from SIV-infected RMs detected endogenous Tat protein, with higher abundance at 16 wpi (Fig. 3G). Tat levels in CSF showed no correlation with either pVLs or cVLs, suggesting that Tat abundance is independent of viral replication (Fig. 3G). Collectively, these findings demonstrate that while productive SIV infection was not established in cerebral tissues during acute infection, soluble viral proteins, particularly Tat, diffused from the CSF into the brain via the PVS. This protein-level infiltration likely contributes to early inflammation and neurodestruction, independent of local viral replication.

### Intracerebroventricular Tat infusion induces neuroinflammation and behavioral deficits

To evaluate the neurodestruction of Tat exposure during acute infection, an intracerebroventricular infusion model was established in mice, in which HIV-1 Tat was administered once a day for five consecutive days. Transcriptomic profiling revealed substantial overlap in immune activation between Tat-infused mice and SIV-infected RMs at early acute stages. Specifically, upregulated GO biological process terms related to immune and defense responses were prominently enriched in both the cerebral cortex (primary somatosensory cortex barrel field, S1BF; medial prefrontal cortex, mPFC) and subcortical nuclei (thalamus, striatum; Fig. 4A and 4B). PPI network analysis of these upregulated immune genes identified *STAT1*, *BST2*, and *DHX58* as central hubs in both brain regions (Fig. 4C), mirroring the immune gene signatures observed in SIV-infected RMs.

**Figure 4.**
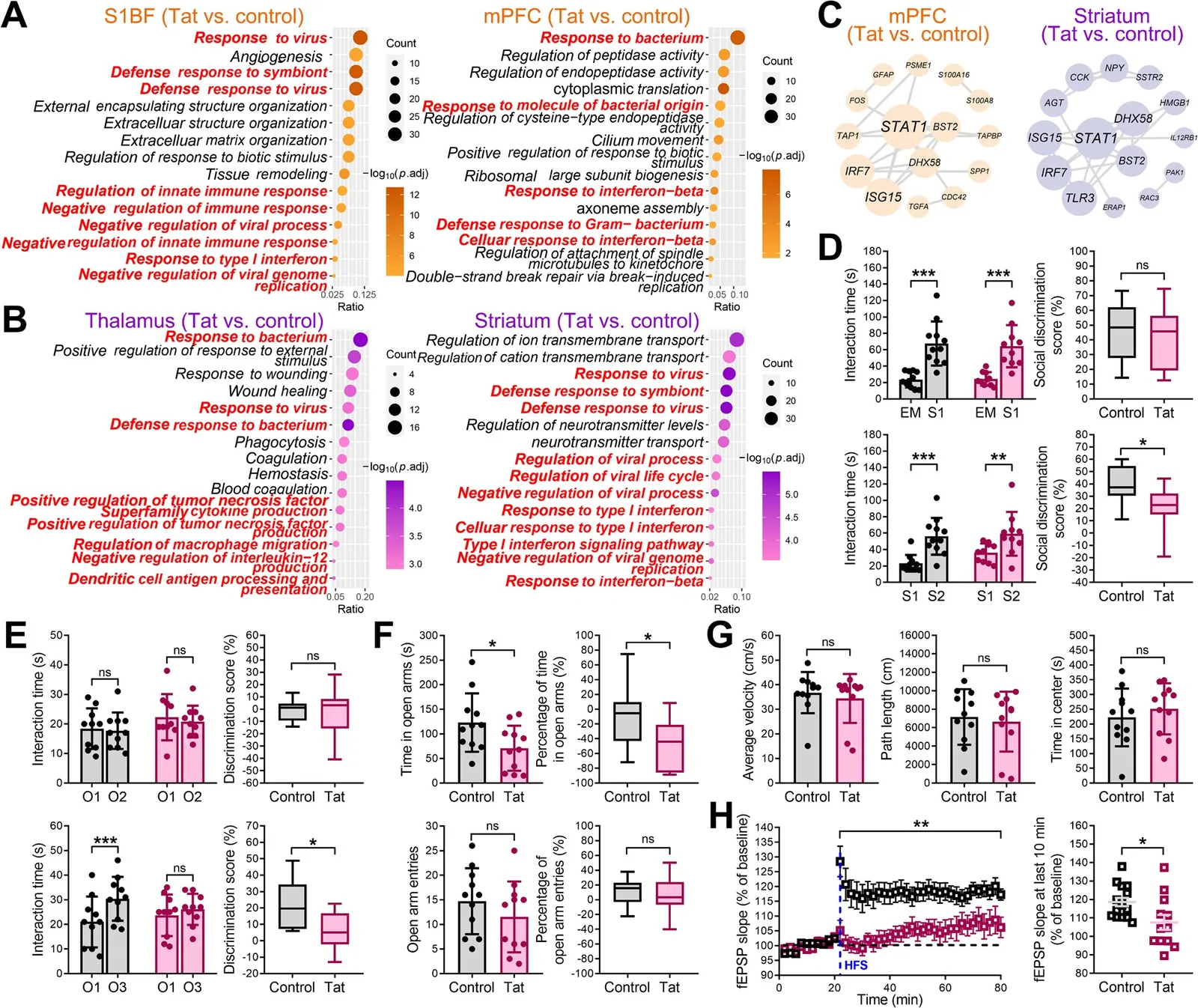
Transcriptomic profiling of brain tissues and behavioral experiments in Tat-infused mice. (A, B) Top 15 GO biological process enriched in upregulated DEGs in the cerebral cortex (A) and subcortical nuclei (B) of mice infused with HIV-1 Tat. (C) PPI networks of the top 15 upregulated immune-related DEGs (ranked by degree) in cerebral cortex (left) and subcortical nuclei (right) of mice infused with HIV-1 Tat. (D, E, F, G) Behavioral experiments in Tat-infused mice, including three-chamber social interaction test (D, *n* = 11 per group; mean ± SD; two-way ANOVA), novel object recognition test (E, *n* = 10 per group; mean ± SD; two-way ANOVA), elevated plus maze test (F, *n* = 11 per group; mean ± SD; unpaired *t*-tests), and open field test (G, *n* = 11 per group; mean ± SD; unpaired *t*-tests). (EM, empty; S1, Stranger mice 1; S2, Stranger mice 2; O1, Object 1; O2, Object 2; O3, Object 3.). (H) Long-term potentiation (LTP)-induced electrophysiological recordings of hippocampal slices from Tat-treated and control groups. (*n* = 15 and 12 for Control and Tat group, respectively; mean ± SEM; two-way ANOVA). (ns, not significant; ^*^P < 0.05, ^**^P < 0.01, ^***^P < 0.001).

To evaluate Tat-induced neurobehavioral changes in the absence of direct viral infection, a series of behavioral assays was conducted. In the three-chamber social interaction test (Fig. 4D), both control and Tat-treated mice demonstrated intact sociability, preferring interaction with a stranger mouse (S1) over an empty chamber. However, during the social novelty phase, while both groups showed a preference for investigating the second stranger mouse (S2) over the now familiar S1 mouse, Tat-treated mice showed a reduced preference, indicating diminished social novelty recognition. Subsequently, the novel object recognition task further revealed impaired short-term memory in the Tat group, which failed to distinguish between familiar and novel objects (Fig. 4E). In the elevated plus maze, Tat-treated mice exhibited increased anxiety-like behavior, spending significantly less time in the open arms than controls (Fig. 4F). The open-field experiment revealed no significant differences in movement speed or distance between groups (Fig. 4G), ruling out gross motor impairment as a confounding factor. Finally, electrophysiological assessment of the hippocampal CA1 region after high-frequency stimulation revealed attenuated long-term potentiation (LTP) in the Tat-infused mice (Fig. 4H), indicating impaired functional connectivity. Collectively, these data demonstrate that HIV-1 Tat introduced into the CSF elicits immune gene expression patterns and central nervous system dysfunction closely resembling those observed in RMs during acute SIV infection. These findings strongly implicate extracellular Tat as a key mediator of early neuroinflammatory injury and cognitive impairment during acute lentiviral infection.

### Regional microglial heterogeneity drives diverse responses to Tat exposure

RNA-seq of healthy RMs and mice revealed physiological up-regulation of immune- and defense-related GO biological process terms in the subcortical nuclei compared to the cerebral cortex (Fig. 5A). PPI network analysis of the top 20 upregulated immune-related DEGs in RMs identified *CD4*, *CD86*, and Toll-like receptors as playing a core role in this regional difference, confirmed by qPCR (Fig. 5B), indicating that heightened immune activity in the subcortical nuclei is a conserved mammalian feature. In SIV-infected RMs, immune and defense-related GO biological process terms were similarly upregulated—in the subcortical nuclei compared to the cerebral cortex at 2 and 16 wpi (Fig. 5C), although the magnitude of regional differences diminished during the later acute stage. Overlaying physiological immune-related DEGs onto the PPI networks from the cerebral cortex and subcortical nuclei (Fig. 2C) revealed that these immune mediators, especially *CD4*, *TLR3*, *TLR4*, *TLR7*, and *MX2*, played a core role in SIV infection-induced neuroinflammation (Fig. 5D), suggesting that regional immune architecture shapes the intensity and composition of the antiviral response. Comparative analysis of fold changes in physiological immune-related DEGs across regions and time points revealed persistent up-regulation in the cerebral cortex during acute SIV infection, whereas expression in the subcortical nuclei remained largely unchanged (Fig. 5E). These findings indicate that baseline regional immune states strongly influence the magnitude and dynamics of acute immune responses. The intrinsically heightened immune capacity of the subcortical nuclei may underlie their propensity for rapid inflammation and neurodestructive processes during acute SIV infection.

**Figure 5.**
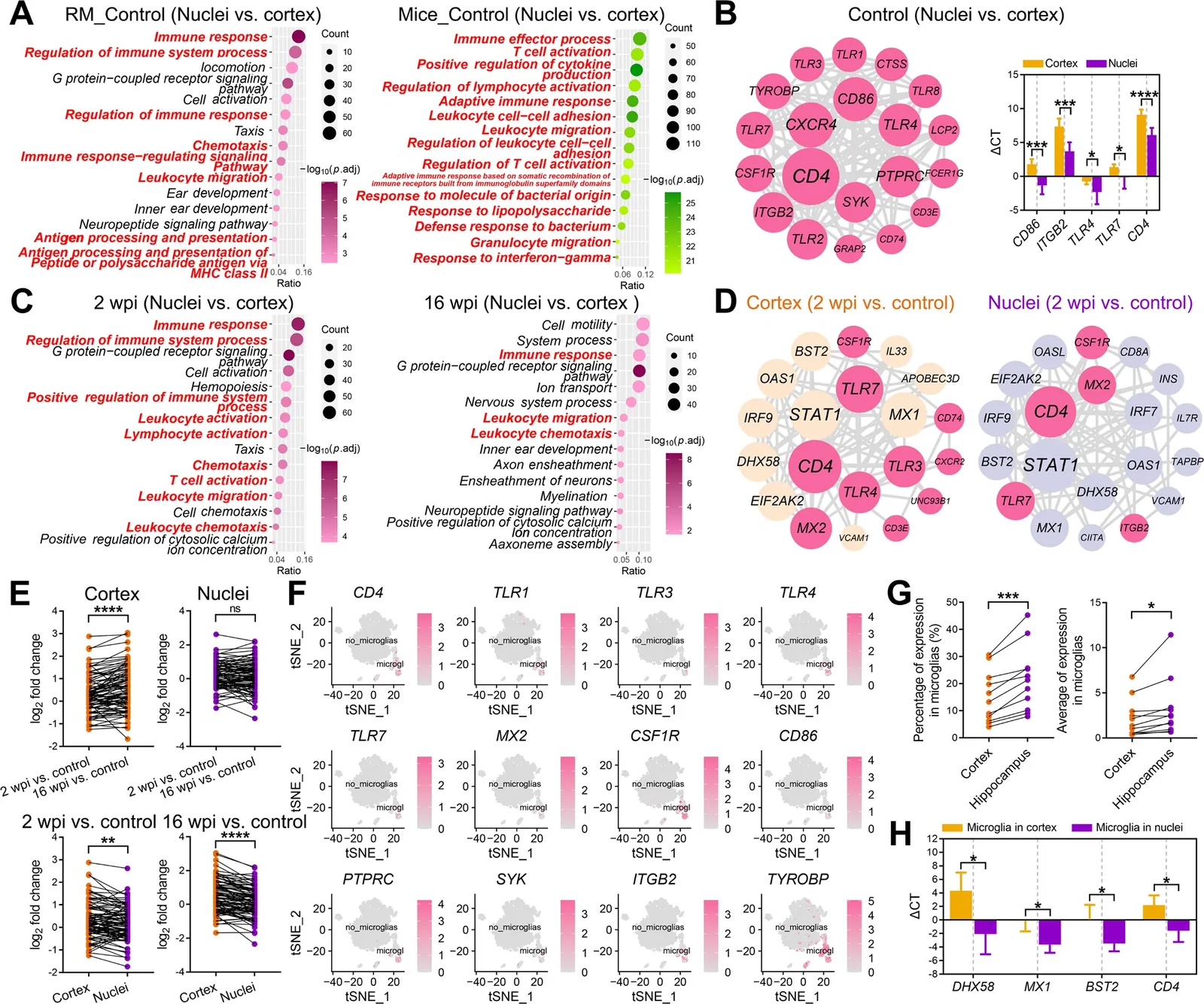
Regional microglial phenotypes and differential reactivity in the cerebral cortex and subcortical nuclei. (A) Top 15 GO biological process enriched in upregulated DEGs for the comparison subcortical nuclei vs. cerebral cortex in healthy RMs (left) and healthy mice (right). (B) PPI networks (left) of the top 20 upregulated immune-related DEGs (ranked by degree) for the comparison subcortical nuclei vs. cerebral cortex in healthy RMs, and qPCR validation of hub gene expression levels (right; *n* = 7 and 6 for Cortex and Nuclei group, respectively; mean ± SD; unpaired *t*-tests). (C) Top 15 GO biological process enriched in upregulated DEGs for the comparison subcortical nuclei vs. cerebral cortex at 2 wpi (left) and at 16 wpi (right). (D) PPI networks of the top 20 upregulated immune-related DEGs (ranked by degree) for the comparison 2 wpi vs. control in the cerebral cortex (left) and subcortical nuclei (right). Red: physiological-immune-related DEGs; orange: independent DEGs in the cerebral cortex; purple: independent DEGs in subcortical nuclei. (E) Fold changes in physiological-immune-related DEGs during acute SIV infection in the cerebral cortex and subcortical nuclei. (*n* = 96 per group; individual values; paired *t*-tests). (F) Expression levels of hub physiological-immune-related DEGs based on single-cell RNA sequencing (scRNA-seq) of the hippocampus. (G) scRNA-seq analysis of positive-cell percentage and average expression of physiological-immune-related DEGs in microglia from the hippocampus and the cerebral cortex. (*n* = 11 per group; individual values; paired *t*-tests). (H) qPCR analysis of hub gene expression levels in microglia from the cerebral cortex and the subcortical nuclei stimulated by HIV-1 Tat *in vitro*. (*n* = 3 per group; mean ± SD; unpaired *t*-tests). (ns, not significant; na, not available; ^*^P < 0.05, ^**^P < 0.01, ^***^P < 0.001, ^****^P < 0.0001).

Finally, single-cell RNA sequencing (scRNA-seq) of cerebral tissues in RMs (Fig. 5F) revealed that the majority of hub physiological immune-related DEGs were enriched in microglia. Comparative profiling of cortical and subcortical regions from the same animal (Fig. 5G) demonstrated a higher proportion and elevated expression of these DEGs in hippocampal microglia relative to those in the cerebral cortex. To directly assess regional microglial responsiveness, cells were isolated from the cortex and subcortical nuclei of a single monkey and exposed to HIV-1 Tat *in vitro*. Tat stimulation induced markedly higher expression of *DHX58*, *MX1*, *BST2*, and *CD4* in microglia from the subcortical nuclei (Fig. 5H). These results indicate that intrinsic regional heterogeneity among microglia governs the magnitude of Tat-induced immune activation, contributing to accelerated and more severe neuroinflammatory damage in the subcortical nuclei during acute SIV infection.

## Discussion

This study provides compelling evidence that acute SIVmac239 infection drives neuroinflammation and neurodestruction in RMs, with five hallmark features: (i) synchronous profiles of plasma and CSF viral loads, with peak levels at 2 wpi; (ii) significant elevation of cytokines in CSF, but not neuronal injury biomarkers, during the early acute stage; (iii) marked increases in structural MRI indices over the course of infection; (iv) localized neuronal degeneration, nuclear dissolution, Nissl body loss, and demyelination; and (v) robust upregulation of immune-, defense-, and infection-related terms, pathways, and genes. Compared with the cerebral cortex, subcortical nuclei exhibited faster and more severe lesions during the acute stage, including more rapid changes in NODDI-derived metrics and histological alterations, higher upregulation of immune-related DEGs, and enrichment of pathways associated with neurodegeneration and synaptic dysfunction. Collectively, these results demonstrate that brain injury occurs as early as the acute stage, with subcortical nuclei sustaining more severe damage than the superficial cerebral cortex (Fig. 6).

**Figure 6.**
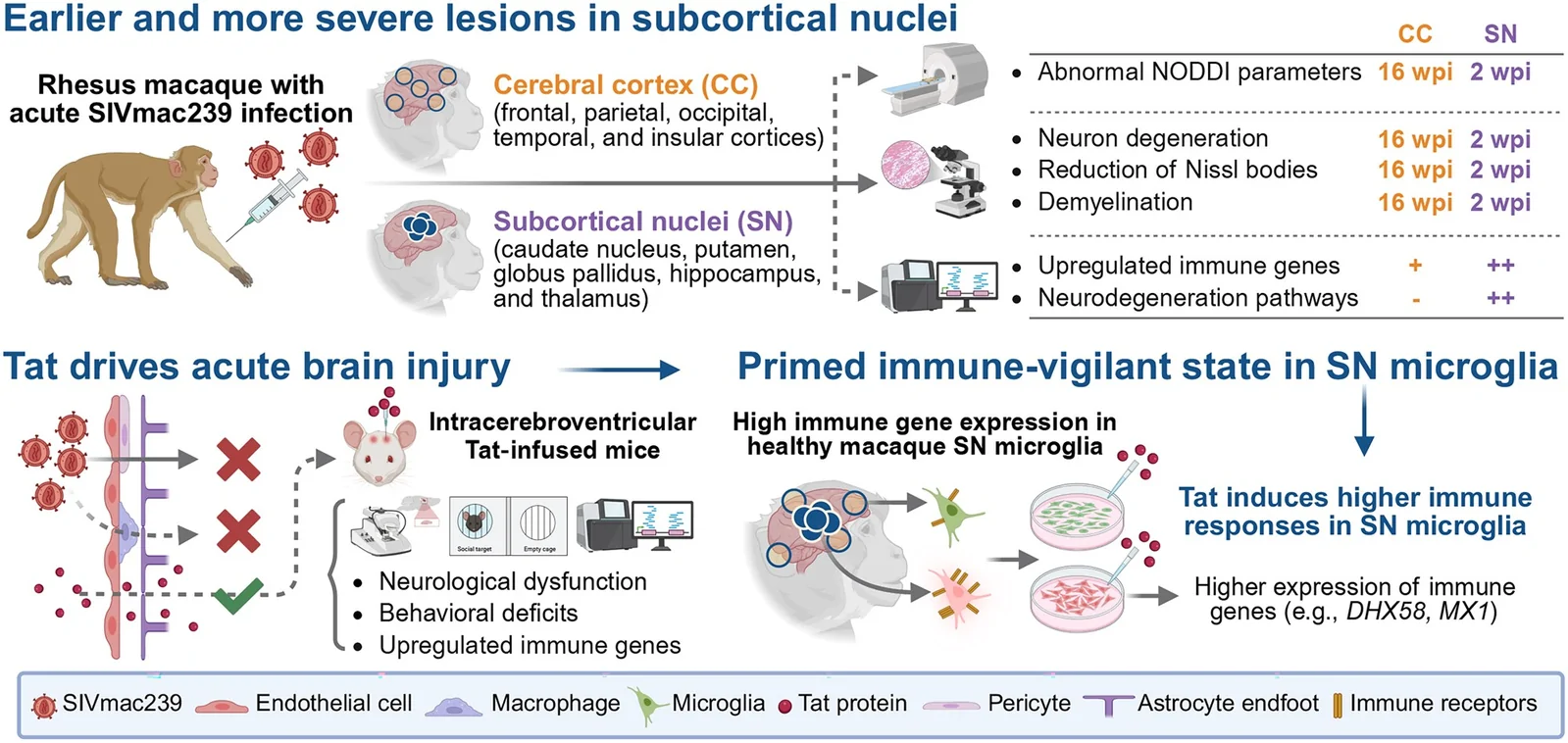
Graphical summary. Acute SIVmac239 infection induces earlier and more severe lesions in the subcortical nuclei than in the the cerebral cortex, as evidenced by abnormal NODDI-derived metrics, neuronal degeneration, Nissl body loss, demyelination, upregulation of immune-related genes, and enrichment of neurodegeneration-associated pathways. Unlike intact virions, the viral protein Tat crosses the blood–brain barrier and diffuses into brain parenchyma via perivascular spaces, driving neuroinflammation and neuronal injury during acute infection. In a mouse model, intracerebroventricular Tat infusion recapitulates key features of acute SIV infection, including behavioral deficits, neurological dysfunction, and upregulation of immune-related genes. Mechanistically, microglia in the subcortical nuclei display a primed, immune-vigilant phenotype at baseline, and respond to Tat exposure with markedly higher expression of immune-related genes compared to their cortical counterparts, underpinning the regional vulnerability. Created in BioRender. Hu, Y. (2026) BioRender.com/g4k0pg1.

The emergence of distinct HIV/SIV variants in the CNS, known as compartmentalization, has been interpreted as evidence for independent viral replication, although this remains contentious, particularly during early infection [24]. However, phylodynamic reconstruction by Sturdevant et al. [25] indicated that independent HIV-1 replication in the CNS occurs only after four months of infection. Furthermore, SIV-infected animal studies suggest that compartmentalization more likely reflects delayed CNS entry of peripherally adapted viral populations rather than *in situ* replication [26], a view supported by Kincer et al. [27] who found that clonally amplified CSF viral lineages correlated with transient blood cell influx after cART interruption, without detectable rebound of macrophage-tropic variants. In this study, viral loads in CSF and plasma exhibited similar magnitudes and temporal trajectories, mirroring strong correlations between cVLs and pVLs in PLWH [28–30]. However, viral RNA, DNA, and proteins were undetectable in all cerebral regions at both 2 and 16 wpi. These results suggest that SIV particles in CSF originate from peripheral blood rather than local brain replication during acute infection, and that delayed establishment of the cerebral viral reservoir does not underlie early neuroinflammatory injury. Several lines of evidence support this conclusion: anti-HIV antibodies appear in CSF at similar levels to plasma but with a 2-week delay [31], antiretroviral intensification fails to ameliorate HAND symptoms [32], and viral RNA-positive cells are rare and confined to perivascular zones in nonaccelerated SIV/SHIV models [33, 34]. Multiplex staining in this study further confirmed that macrophages were strictly restricted to the perivascular space delineated by astrocytic endfeet, arguing against significant parenchymal viral entry via Trojan horse mechanism during the acute phase, as HIV-1 infection cannot be effectively established in astrocytes via cell-free or cell-to-cell routes [22].

Low-molecular-weight viral proteins, including Tat and p17, can traverse the BBB [35, 36]. In this study, evidence from fluorochrome-infused mice demonstrated that low-molecular-weight viral elements (< 20 kDa) in CSF readily disseminate into surrounding brain parenchyma via the PVS, suggesting that viral proteins may induce brain lesions during the acute stage. HIV-1 Tat protein, a key determinant of neuropathogenesis, can elicit profound neuropathological effects through its interaction with microglia, astrocytes, brain microvascular endothelial cells, and neurons, triggering aberrant gene expression, sustained glial activation, proinflammatory signaling, neuronal toxicity, and structural disruption in the brain [37]. Tat was detected in CSF, with levels increasing from 2 wpi to 16 wpi and showing no correlation with plasma or CSF viral loads, mirroring observations in PLWH where circulating Tat is not suppressed by cART [38] and shows no association with CD4^+^ T cell counts, plasma HIV RNA, p24 antigen levels, or anti-Tat antibodies [39]. Intracerebroventricular Tat infusion in mice recapitulated the transcriptional signatures observed in RMs (upregulation of *STAT1*, *BST2*, *DHX58*) and induced behavioral deficits and impaired LTP, strongly implying that Tat may play a central role in cerebral injury and dysfunction during acute HIV/SIV infection.

Extensive distribution of HIV Tat to both cortical and subcortical regions indicated that regional microglial heterogeneity critically shapes differential neuroimmune responses during acute SIV infection. Microglia are permissive to HIV replication, and disruptions in their homeostasis, such as activation, metabolic dysregulation, and apoptosis, have been implicated in the development of HAND [40]. Genome-wide profiling in mice by Grabert et al. [41] demonstrated that microglia in distinct brain regions possess specialized transcriptional programs, particularly within bioenergetic and immunoregulatory pathways, highlighting a more immune-vigilant phenotype in subcortical areas such as the hippocampus. Similarly, scRNA-seq analyses in HIV gp120 transgenic mice revealed that regional microglial diversity underlies differential responses to viral proteins [42]. Consistent with these observations, bulk transcriptomic analyses in both healthy RMs and mice showed pronounced differences in immunoregulatory pathways between the cerebral cortex and subcortical nuclei, with the latter displaying a heightened baseline immune profile. Single-cell transcriptomics further localized key immunoregulatory genes to microglia, reinforcing the presence of a primed immune-vigilant state in subcortical microglia. Functional assays confirmed that HIV-1 Tat stimulation elicited markedly higher induction of *DHX58*, *MX1*, *BST2*, and *CD4* in subcortical-derived microglia, supporting a model in which microglial regional identity governs the intensity of Tat-induced immune activation.

Collectively, these findings establish that acute SIV infection triggers rapid neuroinflammation and neurodestruction in the CNS, with disproportionate severity in the subcortical nuclei. In the absence of a detectable cerebral viral reservoir, HIV/SIV Tat protein reaches deep brain structures and acts as a principal mediator of neuropathogenesis. Subcortical microglia, characterized by an inherently vigilant immunophenotype, respond more rapidly and severely to Tat, leading to region-specific patterns of neural injury distinct from those observed in the cerebral cortex (Fig. 6).

### Research limitations

Several limitations inherent to this proof-of-concept study delineate the interpretive boundaries of the current findings and define priorities for subsequent work. First, only three RMs were included per infected group (2 wpi and 16 wpi). This small sample size may reduce statistical power, and the findings require validation in larger cohorts. Second, SIV strains exhibit differences in tropism, pathogenicity, replication kinetics, and neuroinvasiveness. Given that our study exclusively employed SIVmac239, extrapolation of these findings to other SIV strains, including SIVmac251, should be made with caution. Third, the strong correlation between CSF and plasma viral loads suggests peripheral origin of CSF SIV, but the exact entry route and the source of persistent CSF Tat are not defined. The blood–cerebrospinal fluid barrier (BCSFB) at the choroid plexus, a highly vascularized interface, may allow peripheral proteins or viruses to enter CSF. Unlike the restrictive blood–brain barrier, the BCSFB exhibits developmentally regulated permeability. In this study, SIV and Tat in CSF are unlikely to derive from brain parenchymal replication or simple BBB crossing; thus, the BCSFB likely contributes to acute CNS entry. Direct investigation of this pathway is needed. Fourth, this study focused primarily on Tat. We did not assess whether other neurotoxic viral proteins, such as gp120, Nef, or Vpr, are also present in CSF. Therefore, the possibility that these proteins contribute collectively to acute neural injury cannot be excluded. Subsequent studies should simultaneously detect multiple viral proteins and evaluate their potential synergistic effects.

## Methods

### Animals and ethics statement

Six male RMs (8–10 years old) and cerebral tissues from healthy adult RMs (8–12 years old) were obtained from the Kunming Primate Research Center, Kunming Institute of Zoology, Chinese Academy of Sciences. Fifty male C57BL/6 mice (8 weeks old) were purchased from Sibeifu (Beijing, China). All animals were maintained under standard conditions at 24°C with a 12 h light/dark cycle and provided with free access to food and water. All experimental procedures were conducted in accordance with the institutional guidelines and approved by the Ethics Committee of Kunming Institute of Zoology, Chinese Academy of Sciences (Approval No. IACUC-PE-2021-09-001; date of approval: September 26, 2021).

### SIVmac239 infection and tissue sampling

RMs were intravenously inoculated with 4000 TCID_50_ of SIVmac239 (GenBank accession number: M33262.1). Peripheral blood was collected at 0, 0.5, 1, 2, 4, 6, 8, 10, and 16 wpi, and CSF was collected via lumbar puncture at 0, 2, 6, and 16 wpi. Three animals (IDs: 12053, 12339, and 1105027) were euthanized at 2 wpi and three (IDs: 12373, 12391, and 1105327) at 16 wpi. After transcardial perfusion with precooled phosphate-buffered saline (PBS), tissues including testes, colon, spleen, liver, lungs, lymph nodes, cerebral cortex (frontal, parietal, occipital, temporal, and insular cortices), and sub­cortical nuclei (caudate nucleus, putamen, globus pallidus, ­hippocampus, and thalamus) were collected. All animal experiments were performed under Zoletil 50 anesthesia (Virbac, France).

### SIVmac239 real-time RT-PCR assay

Total RNA in plasma and CSF was extracted using a High Pure Viral RNA Kit (Roche, UK). Tissue RNA and DNA were isolated using TRIzol Reagent (Thermo Fisher Scientific, USA) and a TIANamp Genomic DNA Kit (DP304, Tiangen, China), respectively. Viral RNA and DNA were quantified on a QuantStudio 5 Dx Real-Time PCR System (Thermo Fisher Scientific, USA) using identical primer–probe sets targeting SIVmac239 *gag* gene (Table S2), with a THUNDERBIRD Probe One-Step qRT-PCR Kit (QRZ-101, TOYOBO, Japan) for RNA and Premix Ex Taq^TM^ (RR390A, TAKARA, Japan) for DNA. Standard curves were generated through serial dilutions of the pGEM-4Z-SIV *gag*357 plasmid. The lower limit of detection (LLoD) for plasma and CSF viral RNA was 50 copies/mL. For tissue-associated viral DNA and RNA, the LLoD was determined to be 5 copies per reaction.

### MRI assay

Anesthesia induction and maintenance were achieved using ketamine-HCl (ZMBK, China) and Dexdomitor® (Zoetis, USA), respectively. Whole-brain MRI of RMs was performed at 0, 2, 6, and 16 wpi using a uMR 790 3 T Scanner (United Imaging, China). The imaging protocol included T1, T2, and NODDI sequences. Preprocessed data were segmented using the D99 macaque brain atlas to automatically extract volumes of each brain region [43], and further processed using FSL (FMRIB Software Library, fsl.fmrib.ox.ac.uk/fsl/fslwiki/FSL) and the NODDI MATLAB Toolbox (www.nitrc.org/projects/noddi_toolbox). A total of 46 cortical and 23 subcortical regions were analyzed. Detailed parameters were provided in Supplementary Materials.

### Markers of neuronal injury and inflammation in CSF

Levels of neopterin, NF-L, and YKL-40 in CSF were measured using commercial ELISA kits (Mlbio, China). Albumin levels were determined using an automatic chemistry analyzer (Cobas6000, Roche, Switzerland). Cytokine levels (IP-10, IL-8, GM-CSF, IL-10, IL-12p70, IL-15, IL-6, and TNF-α) were measured using an NHP XL Cytokine Luminex® Performance Premixed Kit (FCSTM21, R&D Systems, USA).

### Histological and immunological staining

Cerebral tissues were fixed in 4% paraformaldehyde, sequentially dehydrated through a graded ethanol series, embedded in paraffin, and sectioned at a thickness of 4 μm. Pathological alterations were histologically assessed using H&E, LFB, and Nissl staining techniques, with subsequent imaging with a brightfield microscope (BX53, Olympus, Japan). Single-target immunofluorescence staining was performed according to standard protocols [44]. Multiplex immunohistochemical staining was implemented using the tyramide signal amplification (TSA) method, as described in our previous study [45]. Detailed information on reagents and analytical methods was provided in the Supplementary Materials.

### Western blot analysis

CSF or tissue homogenate supernatants were mixed with 5× loading buffer (P0286, Beyotime, China), separated by 12.5% sodium dodecyl sulfate–polyacrylamide gel electrophoresis (SDS–PAGE), and transferred onto polyvinylidene fluoride (PVDF) membranes (1620177, Bio-Rad, USA). The membranes were blocked with 5% skimmed milk for 2 h at room temperature, followed by incubation with primary antibodies overnight at 4°C and corresponding horseradish peroxidase (HRP)-conjugated secondary antibodies for 1 h at room temperature. All blots were developed using a chemiluminescent HRP substrate (WBKLS0500, Merck Millipore, USA) and analyzed using ImageJ software. Detailed information on antibodies was provided in Supplementary Materials.

### Intracerebroventricular injection

C57BL/6 mice were anesthetized by intramuscular administration of Zoletil 50 and xylazine (23076-35-9, Aladdin, China). Stereotaxic injections were performed using a stereotaxic apparatus (RWD, China) to target the lateral ventricle, with coordinates from bregma: anteroposterior (AP) − 0.46 mm, mediolateral (ML) ± 1.0 mm, and dorsoventral (DV) − 2.25 mm. Fluorescent tracers and proteins, including 100 nm FluoSpheres (PSOF00100, ZKLM, China), 2000 kDa FITC-dextran (MS0910, MKbio, China), 20 kDa TRITC-dextran (T491224, Aladdin, China), and HIV-1 Tat (ab83353, Abcam, UK), were dissolved in artificial CSF (ACSF, MX0951, MKbio, China). Each mouse received a 4 μL unilateral infusion of ACSF, fluorescent tracer, or HIV-1 Tat (250 ng/μL) into the lateral ventricle through a glass micropipette connected to a syringe pump (Micro 4, USA) at a rate of 800 nL/min. Whole brains were collected following transcardial perfusion with precooled PBS, then sliced with a vibratome (VT1000S, Leica, Germany) for fluorochrome infusion or a freezing microtome (FS800A, RWD, China) for HIV-1 Tat infusion. For repeated Tat administration, bilateral stainless-steel guide cannulas, rather than unilateral glass micropipettes, were implanted into the lateral ventricle region. After a 7-day recovery period, mice were briefly anesthetized with isoflurane (R510-22-16, RWD, China) for 2 min to allow intracerebroventricular injection of 2 μL of ACSF or HIV-1 Tat (25 ng/μL) once daily for 5 days, mimicking the Tat concentration in the CSF of untreated PLWH [38]. The daily Tat dose (50 ng) was calculated based on the measured CSF clearance half-life (about 30 min) of a similarly sized (17 kDa) tracer in awake mice, combined with the mouse CSF volume [46, 47]. Behavioral and electrophysiological experiments were subsequently performed as described in the Supplementary Materials.

### Transcriptome analysis

Bulk RNA-seq libraries were prepared from total RNA (≥ 1 μg) using a NEBNext® Ultra^TM^ RNA Library Prep Kit and sequenced on an Illumina NovaSeq 6000 platform (Novogene, Beijing, China). Clean reads were aligned to the RM reference genome (Mmul_10) and mouse reference genome (C57BL_6NJ_v1). Differential expression analysis was performed using DESeq2 (|log_2_FC| > 2, *P *< 0.05), followed by GO biological process and KEGG enrichment analyses with ClusterProfiler (adjusted *P *< 0.05). For scRNA-seq analysis, the public dataset GSE167319 (cerebral cortex and hippocampus of a healthy RM) was retrieved from the Gene Expression Omnibus (GEO) database. Data were processed using Seurat (v5.0.3); low-quality cells (< 200 or > 2000 genes, > 5% mitochondrial genes) were excluded. Microglial populations were identified using *P2RY12* and *CSF1R* markers. Detailed parameters and procedures are provided in the Supplementary Materials.

### HIV-1 Tat stimulation *in vitro*

Primary microglia were isolated from brain tissues (frontal, parietal, occipital cortices, basal ganglia, hippocampus, and thalamus) of a healthy RM (No. 19313) via mechanical dissociation followed by fluorescence-activated cell sorting (FACS) using anti-CD11b antibody (BioLegend, USA). Isolated cells were cultured in DMEM/F12 with 15% FBS for 12 h, then stimulated with HIV-1 Tat (10 ng/mL) for 24 h. Total RNA was extracted using TRIzol Reagent, and the expression of *DHX58*, *MX1*, *BST2*, and *CD4* was quantified by real-time PCR with TB Green® Premix Ex Taq^TM^ II (RR820A, TAKARA, Japan). Relative expression was normalized to *RPL13A*. Primer sequences are listed in Table S2.

### Statistical analysis

Two-way analysis of variance (ANOVA) was used to analyze data from the novel object recognition test, three-chamber social interaction test, electrophysiological experiments, and MRI experiments. Paired *t*-tests were employed to evaluate differences in CSF neurodestructive and inflammatory markers, fold changes in DEGs, and specific gene expression in scRNA-seq data. Unpaired *t*-tests were applied to analyses of open field and elevated plus maze tests, histological and immunofluorescence results, and relative gene expression in microglia between cortical and subcortical regions. All statistical analyses were performed using GraphPad Prism v8 (GraphPad, USA). All results are presented as mean ± standard deviation (SD) from at least three repeats, except for electrophysiological results, which are presented as mean ± standard error of the mean (SEM). *P *< 0.05 was considered statistically significant.

## Supplementary Material

lnag033_Supplementary_Data

## Data Availability

All data from this study are available from the corresponding authors upon reasonable request. The sequencing data were successfully deposited in the GSA under accession code CRA023654.
