# Supplementary material for: Brain region-specific lesions in the early AIDS monkey model
Source: Life Med. 2026 Sep 16;5(5):lnag033. doi: 10.1093/lifemedi/lnag033 (PMC13694104; doi:10.1093/lifemedi/lnag033)
Supplement: Supplementary file 1 [file lnag033_supplementary_data.zip › Supplementary_Files_(LIFEMED-2026-030)-.docx]

**Brain region-specific lesions in the early AIDS monkey model**

Tian-Zhang Song^1,2,#^, Yan Hu^1,2,6,#^, Shi-Zhe Wang^1,3,6,#^, Huan-Zhi Chen^6^, Bei-Bei Tang^1,2,4^, Jin-Nan Li^1,3^, Qing Li^1,2^, Rong-Yao Huang^1,5^, Li-Rong Zhang^1,2,4^, Lin Xu^1,3,*^, Yong-Tang Zheng^1,2,*^

**Supplementary materials**

***Magnetic resonance imaging (MRI) parameters***

The acquisition parameters were as follows: for T1: repetition time (TR) 13.01 ms, echo time (TE) 5.6 ms, inversion time (TI) 880 ms, flip angle (FA) 8°, number of averages 2, matrix size 192 × 160 × 150, voxel size 0.5 × 0.5 × 0.5 mm^3^; for T2: TR 3400 ms, TE 369.48 ms, and FA 59°, number of averages 2, matrix size 192 × 160 × 150, voxel size 0.5 × 0.5 × 0.5 mm^3^; and for neurite orientation dispersion and density imaging (NODDI): TR 5900 ms, TE 76 ms, FA 90°, b-values 1500 g/mm² and 3000 g/mm², number of averages 2, matrix size 96 × 96 × 82, voxel size 1.0 × 1.0 × 1.0 mm^3^. 46 cortical regions included 7op, 23b, 13m, 12m, 8Bd, 8Bm, 8Bs, 9d, 11l, 13b, 13l, 14r, 23a, 13a, 24a', 29, 9m, 31, 36c, 46v, 24b, 1-2, 30, 24b', 24a, 24c', 23c, 14c, 35, 32, 10o, 44, 24c, 12r, 25, 11m, 36p, 46d, 36r, 12l, 12o, 3a/b, F3, F6, F7, and F4. 23 subcortical regions included CE, Ld, AAA, Lv, ABmc, Bi, ABpc, ME, Bmc, Bpc, CA1, CA2, CA3, CA4, DG, PI, PL, PM, STN, Striatum, Claustrum, GPe/GPi, and SNr/c.

***Immunological Staining***

Paraffin-embedded sections were incubated with primary antibodies, including mouse anti-ZO-1 antibody (33-9100, Invitrogen, USA), mouse anti-occludin antibody (33-1500, Invitrogen, USA), rabbit anti-CD163 antibody (ab182422, Abcam, UK), rabbit anti-GFAP antibody (80788T, Cell Signaling, USA), and rabbit anti-CD3 antibody (ab16669, Abcam, UK). Secondary antibodies included goat anti-mouse IgG H&L (Alexa Fluor^®^ 555, ab150114, Abcam, UK), goat anti-rabbit (HRP, BS13278, Bioworld, USA), and goat anti-mouse IgG (HRP, M21001, Abmart, China). Tyramine with iFluor 488 (45100, AAT Bioquest, USA), Cy3 (11065, AAT Bioquest, USA), or Cy5 (11061, AAT Bioquest, USA) was used in the tyramide signal amplification (TSA) system. Frozen cerebral sections from mice administered a single infusion of Tat were incubated with mouse anti-HIV-1 Tat antibody (ab42359, Abcam, UK) and stained using the TSA method. A Mouse-on-Mouse Polymer IHC Kit (ab269452, Abcam, UK) was used to block endogenous mouse antibodies in the tissue, preventing the non-specific binding of anti-mouse secondary antibodies.

***Antibodies for Western blot***

The primary antibodies included rabbit anti-Tat polyclonal antibody (generated in-house), rabbit anti-P55 polyclonal antibody (DPAB3985, Creative Diagnostics, USA), and rabbit anti-GAPDH antibody (AF7021, Affinity, USA). The secondary antibody was goat anti-rabbit antibody (BS13278, Bioworld, USA).

***Three-chamber social interaction test***

A custom-built rectangular box (60 cm × 40 cm × 22 cm) was divided into three interconnected chambers. Each subject mouse was allowed to freely explore the apparatus for 10 min. For the sociability phase, a stranger mouse (S1) was placed inside a wire cage in the left or right chamber, while the opposite cage remained empty, and the subject mouse was allowed to freely explore for another 10 min. Time spent sniffing either the cage containing S1 or the empty cage was recorded. After 10 min of isolation in the middle chamber, the subject mouse was allowed to freely explore for another 10 min, during which a new stranger mouse (S2) was placed in the empty cage to evaluate social novelty preference. Time spent sniffing S1 or S2 was recorded. Both S1 and S2 were unfamiliar to the subject mouse prior to testing.

***Novel object recognition*** ***test***

The novel object recognition test was performed as described previously [1]. In brief, following habituation to the testing arena, each subject mouse was allowed to freely explore two identical Lego objects (O1 and O2) for 10 min. After 10-min isolation interval, the subject mouse was reintroduced and allowed to freely explore for another 10 min, during which one familiar object (O1) and one novel plaster object (O3) were presented. Time spent sniffing O1, O2, or O3 was recorded. The preference index was calculated as [(time exploring new object) – (time exploring familiar object)] / [(time exploring new object) + (time exploring familiar object)].

***Elevated plus maze test***

Each subject mouse was placed on an elevated plus maze (ENV-560A, Med Associates Inc., USA) and allowed to explore freely for 5 min. Time spent in the open and closed arms was recorded with a stopwatch.

***Open field test***

Each subject mouse was placed in an open field device (MED-OFAS-MSU, Med Associates Inc., USA) and allowed to freely explore for 15 min. Locomotor activity data were collected and analyzed using Activity Monitor software (v7).

***Electrophysiological recordings***

Hippocampal slices were prepared from mice repeatedly infused with HIV-1 Tat, as described previously [2]. In brief, brains were rapidly extracted and immersed in artificial cerebrospinal fluid (ACSF) continuously oxygenated with a gas mixture of 95% oxygen and 5% carbon dioxide. Coronal slices (350 μm) were sectioned using a vibratome and transferred to a recording chamber perfused with standard ACSF at room temperature. Slices were positioned on the stage of a microscope (600-FN, Nikon, Japan) for electrophysiological recordings. Bipolar stimulating electrodes were constructed from a pair of Teflon-coated 90% platinum/10% iridium wires. Recording electrodes (1–3 MΩ) were pulled from borosilicate glass capillaries (1.5 mm outer diameter, 0.84 mm inner diameter, World Precision Instruments, USA) using a Brown-Flaming micropipette puller (P-97, Sutter Instruments Company, USA) and filled with ACSF. Field excitatory postsynaptic potentials (fEPSPs) in the Schaffer collateral (SC)-CA1 pathway were recorded in the stratum radiatum of the dorsal CA1 region of the hippocampus via SC stimulation. GABA-dependent inhibitory transmission was not pharmacologically blocked during fEPSP recordings. For long-term potentiation (LTP) induction, a stable baseline was established with a stimulation strength set to elicit a response equivalent to approximately 40%–50% of the maximal fEPSP slope, maintained for at least 20 min at 0.033 Hz. LTP was then induced by high-frequency stimulation consisting of three 1-sec trains at 100 Hz, separated by 20-sec intervals, at the same stimulus intensity as for baseline recording. All data were recorded using pCLAMP v10 software (Molecular Devices, USA), and signals were collected using MultiClamp 700B and Digidata 1440A (Molecular Devices, USA).

***Bulk RNA sequencing (RNA-seq)***

RNA library construction and sequencing were carried out by Novogene (Beijing, China). In brief, total RNA (≥ 1 μg per sample) was used for library preparation with a NEBNext^®^ Ultra^TM^ RNA Library Prep Kit for Illumina^®^ (NEB, USA), following the manufacturer’s recommendations. Sequencing was conducted on the Illumina NovaSeq 6000 platform (Illumina, USA). Clean data were obtained by removing reads containing adapters, > 10% poly-N bases, and > 50% low-quality (Q ≤ 5) bases. Reference genomes for rhesus macaques (RMs, *Macaca_mulatta*, Mmul_10, release-104) and mice (*Mus_musculus_c57bl6nj*, C57BL_6NJ_v1, release-110) were downloaded from Ensembl. Differential gene expression analysis was performed using DESeq2, calculating log_2_ fold changes and corresponding statistical significance. Differentially expressed genes (DEGs) were annotated with fold change > 2 and *P* < 0.05. Functional enrichment analysis of DEGs was conducted with the ClusterProfiler package in R (v 4.2.3). Gene Ontology Biological Process (GO biological process) and Kyoto Encyclopedia of Genes and Genomes (KEGG) enrichment analyses were performed based on genome-wide annotation for monkeys (org.Mmu.eg.db) and mice (org.Mm.eg.db). Significant enrichment of gene sets was based on an adjusted *P*-value (*p*.adj) < 0.05. Immune-related DEGs were identified using the ImmPort database. Protein-protein interaction (PPI) networks for DEGs were generated using the STRING web platform for visualization and interaction mapping.

***Single-cell RNA sequencing (scRNA-seq)***

The scRNA-seq dataset (accession number GSE167319) was retrieved from the Gene Expression Omnibus (GEO) database (www.ncbi.nlm.nih.gov/geo/) [3]. The dataset comprised samples from the cerebral cortex (accession number GSM5101895) and hippocampus (accession number GSM5101900) of a healthy RM (No. 16271). Data processing and analysis were performed using the Seurat package (v5.0.3) in R. Briefly, low-quality cells and potential doublets expressing fewer than 200 or more than 2000 genes, or containing over 5% mitochondrial gene content, were excluded from downstream analysis. Expression values were normalized by total counts per cell and log-transformed. Highly variable genes were identified using the FindVariableFeatures function, followed by linear scaling of the dataset. The ScaleData function was applied to remove unique molecular identifier counts and mitochondrial gene percentages. Dimensionality reduction was achieved by principal component analysis (PCA) on the scaled matrix. Cell clusters were identified using the FindClusters function based on shared nearest neighbor modularity optimization, with clustering resolution tested between 0.1 and 1.0. Microglial populations were distinguished from non-microglial cells using expression markers *P2RY12* and *CSF1R*. DEGs, including *CD4*, *TLR1*, *TLR3*, *TLR4*, *TLR7*, *MX2*, *CSF1R*, *CD86*, *PTPRC*, *SYK*, *ITGB2*, and *TYROBP*, were visualized using t-distributed stochastic neighbor embedding (t-SNE).

**References**

1.	Leger M, Quiedeville A, Bouet V, et al. Object recognition test in mice. Nat Protoc. 2013;8(12):2531-2537.

2.	Chai AP, Chen XF, Xu XS, et al. A temporal activity of CA1 neurons underlying short-term memory for social recognition altered in PTEN mouse models of autism spectrum disorder. Front Cell Neurosci. 2021;15:699315.

3.	Jiao L, Yang Y, Yu W, et al. The olfactory route is a potential way for SARS-CoV-2 to invade the central nervous system of rhesus monkeys. Signal Transduct Target Ther. 2021;6(1):169.

**Supplementary Table 1**. Characteristics of cerebral tissues for bulk RNA sequencing.

| Group | ID | Cerebral samples | | | | | | | | | |
| --- | --- | --- | --- | --- | --- | --- | --- | --- | --- | --- | --- |
|  |  | Frontal cortex | Parietal cortex | Temporal cortex | Occipital cortex | Insular cortex | Thalamus | Caudate nucleus | Putamen | Globus pallidus | Hippocampus |
| Control | 13483 |  | √ | √ |  |  |  | √ | √ | √ |  |
|  | 13391 | √ | √ | √ |  |  |  |  | √ |  | √ |
|  | 1104215 | √ | √ | √ |  | √ |  | √ | √ | √ |  |
|  | 09076 | √ | √ | √ | √ | √ | √ | √ | √ | √ | √ |
|  | 12426 | √ | √ | √ | √ | √ | √ | √ | √ | √ | √ |
| 2 wpi | 12053 | √ | √ | √ | √ | √ | √ | √ | √ | √ | √ |
|  | 12339 | √ | √ | √ | √ | √ | √ | √ | √ | √ | √ |
|  | 1105027 | √ | √ | √ | √ | √ | √ | √ | √ | √ | √ |
| 16 wpi | 12373 | √ | √ | √ | √ | √ | √ | √ | √ | √ | √ |
|  | 12391 | √ | √ | √ | √ | √ | √ | √ | √ | √ | √ |
|  | 1105327 | √ | √ | √ | √ | √ | √ | √ | √ | √ | √ |

**Supplementary Table 2**. List of PCR primers and probes used in this study.

| **Gene** | **Sequence** |
| --- | --- |
| SIV-*gag* (Forward primer) | 5'-TCGGTCTTAGCTCCATTAGTGCC-3' |
| SIV-*gag* (Reverse primer) | 5'-GCTTCCTCAGTGTGTTTCACTTTC-3' |
| Probe | 5'-CTTCTGCGTGAA TGCACCAGATGACGC-3' |
| *RPL13A* (Forward primer) | 5'-GGCTAAACAGGTACTGCTGGG-3' |
| *RPL13A* (Reverse primer) | 5'-AGGAAAGCCAGGTACTTCAACTT-3' |
| *STAT1* (Forward primer) | 5'-ACTCTGTGCATGCAACCTCG-3' |
| *STAT1* (Reverse primer) | 5'-ATACCACTGAGACATCCTGCC-3' |
| *DHX58* (Forward primer) | 5'-ATGGAGCTGCGGCCCTAC-3' |
| *DHX58* (Reverse primer) | 5'-ACCAGGTGCACCCTGTTGA-3' |
| *MX1* (Forward primer) | 5'-GACCACCCACATTTCAGGGAT-3' |
| *MX1* (Reverse primer) | 5'-CGATGAGCTCGCTGGTAAGT-3' |
| *BST2* (Forward primer) | 5'-GCAGTGATGGAGTGTCGCAA-3' |
| *BST2* (Reverse primer) | 5'-AAGCCATTAGGGCCATCACA-3' |
| *CD4* (Forward primer) | 5'-CTGACCTGTACAGCTTCGCA-3' |
| *CD4* (Reverse primer) | 5'-GCTCAGCTTGGATGGACCTTT-3' |
| *TLR7* (Forward primer) | 5'-TTGGCACCTCTCATGCTCTG-3' |
| *TLR7* (Reverse primer) | 5'-GTGTCCACACTGGAAACATCATT-3' |
| *TLR4* (Forward primer) | 5'-GGGACTCTGATCCCAGCCAT-3' |
| *TLR4* (Reverse primer) | 5'-GTTAGGAACCACCTCCACGC-3' |
| *ITGB2* (Forward primer) | 5'-GACTGAAGCAGGCGCGAAG-3' |
| *ITGB2* (Reverse primer) | 5'-GCACTCCTGAGAGAGGACACA-3' |
| *CD86* (Forward primer) | 5'-TCCAAGTATATGGGCCGCAC-3' |
| *CD86* (Reverse primer) | 5'-GGTTGACTGAAGTTAGCAAGCA-3' |
| *MX2* (Forward primer) | 5'-AATCGTAACCAGGTGTCCGC-3' |
| *MX2* (Reverse primer) | 5'-TGACGTTCTGGGCTTTGTGT-3' |
| *IRF9* (Forward primer) | 5'-CTTATCACAGTGAAGATGGAGCA-3' |
| *IRF9* (Reverse primer) | 5'-AAGAGGACAGGTCAATCGTG-3' |
| *EGFR* (Forward primer) | 5'-TCTTCGGGAAGCAGCTATGC-3' |
| *EGFR* (Reverse primer) | 5'-CGTGCCTTGGCAAACTTTCT-3' |
| *TLR3* (Forward primer) | 5'-GGGACTTTGAGGCAGGTGTT-3' |
| *TLR3* (Reverse primer) | 5'-CGGCATGATGTACCTTGAATCTTTT-3' |
| *ICAM* (Forward primer) | 5'-CACTGGGGGACCAGAAGTTG-3' |
| *ICAM* (Reverse primer) | 5'-GGTGCCGGAAAGCTGTAGAT-3' |
| *CSF1R* (Forward primer) | 5'-GCCACACTGATAGGTGCGAT-3' |
| *CSF1R* (Reverse primer) | 5'-GGCCCTGCACTCATAGGTTT-3' |
| *FGF2* (Forward primer) | 5'-AAGCGGCTGTACTGCAAAAAC-3' |
| *FGF2* (Reverse primer) | 5'-TTGAAGTTGTAATTTGATGTGAGGG-3' |
